# Protein O-GlcNAcylation in multiple immune cells and its therapeutic potential

**DOI:** 10.3389/fimmu.2023.1209970

**Published:** 2023-08-22

**Authors:** Huanhuan Cai, Wei Xiong, Haoyan Zhu, Qiongxin Wang, Shi Liu, Zhibing Lu

**Affiliations:** ^1^ Department of Cardiology, Zhongnan Hospital of Wuhan University, Wuhan, China; ^2^ Institute of Myocardial Injury and Repair, Wuhan University, Wuhan, China; ^3^ State Key Laboratory of Virology, Modern Virology Research Center, College of Life Sciences, Wuhan University, Wuhan, China

**Keywords:** OGT, OGA, O-GlcNAcylation, innate immunity, immune cells

## Abstract

O-GlcNAcylation is a post-translational modification of proteins that involves the addition of O-GlcNAc to serine or threonine residues of nuclear or cytoplasmic proteins, catalyzed by O-GlcNAc transferase (OGT). This modification is highly dynamic and can be reversed by O-GlcNAcase (OGA). O-GlcNAcylation is widespread in the immune system, which engages in multiple physiologic and pathophysiologic processes. There is substantial evidence indicating that both the hexosamine biosynthesis pathway (HBP) and O-GlcNAcylation are critically involved in regulating immune cell function. However, the precise role of O-GlcNAcylation in the immune system needs to be adequately elucidated. This review offers a thorough synopsis of the present research on protein O-GlcNAcylation, accentuating the molecular mechanisms that control immune cells’ growth, maturation, and performance *via* this PTM.

## Introduction

**Figure 1:**
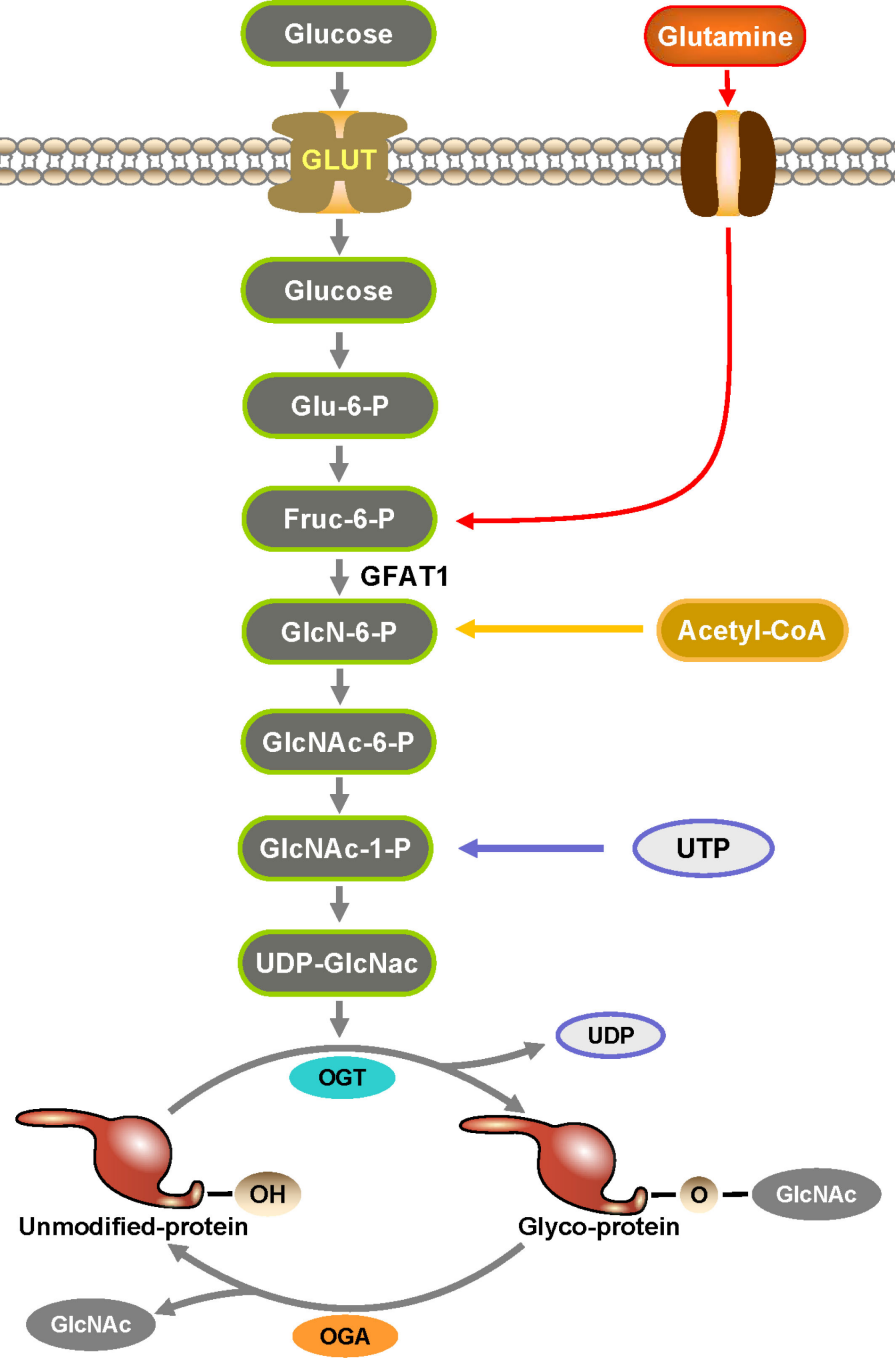
Hexosamine biosynthesis pathway (HBP) is an essential part in the formation of O-GlcNAc. HBP is a branch of glucose metabolism where the vast majority of glucose is used for energy production, glycogen synthesis, and gluconogenesis into fats and proteins, while a small percentage of glucose is used by HBP to produce UDP-GlcNAc. UDP-GlcNAc is catalyzed by OGT to transfer O-GlcNAc to the serine and threonine sites of proteins to complete the O-GlcNAcylation of proteins.

**Figure 2:**
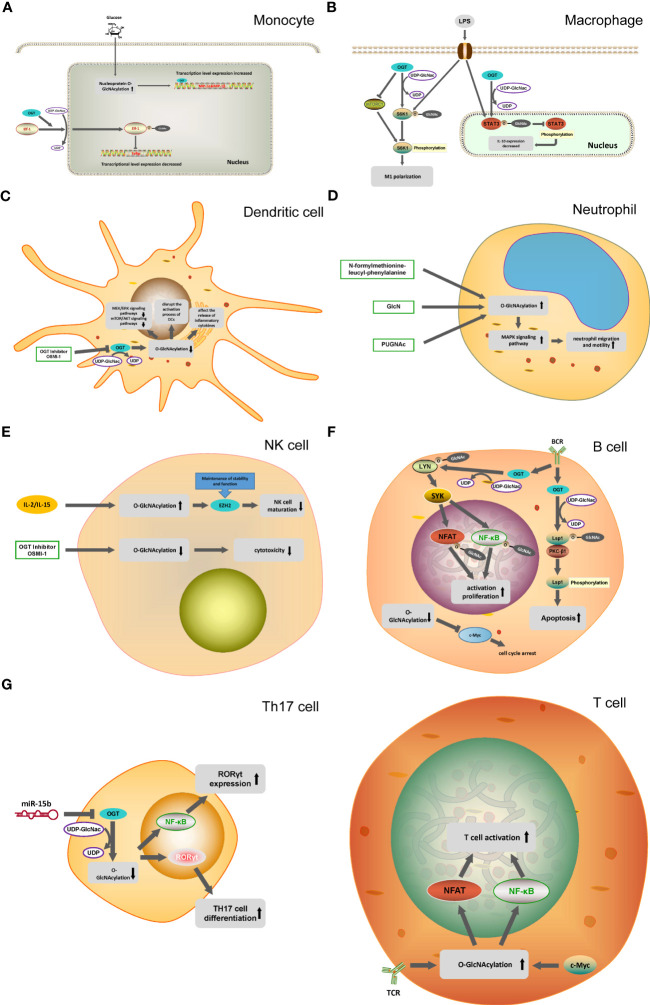
O-GlcNAcylation in different immune cells. **(A)** In monocytes, O-GlcNAcylation helps translocation of the transcription factor Elf-1 into the nucleus and inhibits Tolip transcription. OGT can mediate the transcription of MIP-1 cytokine induced by glucose. **(B)** OGT inhibit mTORC1/S6K1 signaling pathway and the proinflammatory differentiation of macrophages. OGT-mediated O-GlcNAcylation of STAT3 in macrophages inhibit the phosphorylation of STAT3 and the expression of IL-10. **(C)** OGT inhibitor OSMI-1 disrupts the MEK/ERK signaling pathway and mTOR/AKT signaling pathway through the inhibition of O-GlcNAcylation in DC cells, and affects the activation and release of inflammatory factors in DC cells. **(D)** N-formylmethionine-leucyl-phenylalanine, GlcNH, PUGNAc promote the O-GlcNAcylation of neutrophils and activate the MAPK signaling pathway, ultimately promoting the migration and movement of neutrophils. **(E)** IL-2 and IL-15 can cause the O-GlcNAcylation of NK cells to increase, reducing the activity of EZH2 to inhibit the maturation of NK cells. **(F)** In B cells, OGT promote the phosphorylation process of Lsp1 by mediating the O-GlcNAcylation of Lsp1, thus inducing apoptosis. O-GlcNAcylation of Lyn plays a key role in BCR-mediated B-cell activation. Decreased O-GlcNAcylation in B cells down-regulates c-Myc expression, leading to cell cycle arrest. **(G)** T cell activation is dependent on the O-GlcNAcylation of NFAT and NF-κB. miR-15b inhibit the expression of OGT and induce the decline of O-GlcNAcylation, which ultimately leads to the enhancement of Th17 cell differentiation.

Hart and Torres reported the initial identification of protein O-GlcNAcylation when they studied the changes on cell surface proteins of lymphocytes, and they found that most of these proteins are located intracellularly (1–3). O-GlcNAcylation is among the most commonly occurring post-translational modifications (PTM) and is a dynamic PTM analogous to phosphorylation (4). O-GlcNAcylation affects a protein’s structural composition and stability and is crucial for signal transduction, metabolic pathways, immune regulation, oncogenesis and metastasis (5–9). In this process, O-GlcNAc transferase (OGT) catalyzes the addition of monosaccharides, while O-GlcNAcase (OGA) facilitates their removal (10, 11). O-GlcNAcylation of proteins is mediated by the hexosamine biosynthetic pathway, which accounts for approximately 2-5% of glucose utilization (**Figure 1**). Despite the intricate network of cells and proteins within the immune system, the current study has confirmed that O-GlcNAcylation has been shown to promote the growth and activation of T and B cells (12, 13). O-GlcNAcylation has also been shown to exert regulatory effects on inflammation and the innate antiviral immune response mediated by macrophages (14, 15). Additionally, it has been found to enhance the functionality of activated neutrophils while suppressing the activity of natural killer cells (2). **Figure 2** outlines the biological functions of O-GlcNAcylation in the different immune cells mentioned in this review.

## Innate immunity

Innate immunity is also known as nonspecific immunity. The primary function of innate immunity is anti-infection, which is the primary defense system against the invasion of pathogens (16, 17). Upon encountering infection or injury, innate immune cells recognize pathogen-associated molecular patterns (PAMPs), which trigger inflammation and initiate a sequence of inflammatory responses (18, 19). Innate immune cells, such as circulating leukocytes including monocytes and neutrophils, as well as macrophages, natural killer cells (NK cells), and dendritic cells (DCs), are vital in this process (17). Innate immune cells exert their regulatory function through O-GlcNAcylation on their surface and its corresponding receptors. The modification of proteins involved in the NF-κB pathway and other inflammation-associated signaling pathways by O-GlcNAcylation is vital for regulating the functionality of innate immune cells (20). In this section, we illustrate O-GlcNAcylation alteration in innate immune cells (monocytes, macrophages, dendritic cells, neutrophils and natural killer cells) and their subsequent reflection and progress.

## O-GlcNAcylation in monocytes

Human monocytes make up approximately 10% of peripheral leukocytes. In comparison, mice monocytes account for 4% (21). Monocytes originate from the common myeloid progenitor (CMP) differentiated from hematopoietic stem cells (HSCS). They are considered to be the precursor of macrophages and dendritic cells (21, 22). The latest research suggests that there are two distinct differentiation monocyte pathways: (1) CMP → Granulocyte-monocyte progenitor (GMP) → monocytes, (2) CMP → Monocyte-Dendritic cell progenitor (MDP) → monocytes (23). Monocytes can also be considered immature macrophages. Distinct developmental pathways from blood monocytes give rise to diverse subsets of human macrophages (24). One study demonstrated that O-GlcNAc modification is essential for the nuclear translocation of the transcription factor Elf-1, which suppresses Tollip gene transcription in monocytes (25). Zhou W et al. indicated that glucosamine could recruit Ly6C^low^ monocytes and promote recovery from cardiac ischemia in a STAT1 and O-GlcNAcylation dependent manner (26). Currently, it is known that prolonged exposure of monocytes to high levels of glucose can induce a pro-inflammatory phenotype, but the precise role of O-GlcNAcylation in regulating this process in monocytes remains unclear (27). Kato S et al. showed that OGT is essential for MIP-1 genes expression induced by glucose in monocytes, with O-GlcNAcylation and OGT recruitment observed at the MIP-1α promoter. However, HCF-1 was not detected at the MIP-1α promoter in the absence of its activation (28). At present, more evidence needed to reveal the direct regulation of glucose metabolism and pro-inflammatory phenotype by O-GlcNAcylation.

## O-GlcNAcylation in macrophages

Macrophages have diverse origins and are widely distributed throughout various tissues (29). One subset of macrophages is derived from the embryonic yolk sac and fetal liver (30), and another subset is derived from monocytic differentiation (31, 32). As a crucial cell type in innate immunity, macrophages participate in the elimination of damaged and aging cells, and the stimulation of adaptive immunity through antigen presentation. In addition to phagocytosis and immune prevention, they are also involved in tissue metabolism and the maintenance of tissue homeostasis (33, 34). O-GlcNAcylation of p65 near the S536 site can enhance the binding of p65 to IκB, promoting macrophage conversion to the M2 phenotype and alleviating the inflammatory response (35). Yang X et al. suggested that overnutrition triggers the O-GlcNAc signaling pathway in macrophages, leading to the suppression of proinflammatory activation and protecting against obesity and metabolic dysfunction caused by dietary factors (10). Moreover, a study showed that OGT could hinder macrophage proinflammatory activation by repressing the mTORC1/S6K1 signaling pathway (36). The modulation of iNOS in macrophages is pivotal for maintaining the immune system and blocking the O-GlcNAc cycling mechanism leading to an elevation in pro-inflammatory cytokines including IL-1β, IL-6, and IL-12 (37). In human carotid atherosclerotic plaques, a significant number of M1 macrophages (iNOS^+^) could be modified by O-GlcNAc glycosylation. In contrast, M2 macrophages (Arg-1^+^) only occasionally demonstrate O-GlcNAc positivity. A study indicated that elevated blood sugar levels might induce a shift in macrophage polarization towards an alternative phenotype through increased O-GlcNAcylation, which may promote immune evasion. They showed that increased glucose flux promotes tumor growth and immune escape by upregulating O-GlcNAcylation in tumor-associated macrophages (TAMs), leading to a shift in macrophage polarization towards an M2-like phenotype (38). Wen H et al. demonstrated that O-GlcNAcylation of STAT3 on threonine 717 (T717) exerts a negative regulation on its phosphorylation and modulates gene expression in macrophages (39). Shevde LA et al. suggested that tumorigenic Hedgehog (Hh) signaling is vital for programming the metabolic circuitry that regulates macrophage polarization. The study showed that suppression of the Hh pathway in M2 macrophages decreased UDP-GlcNAc biosynthesis flux pathway. This led to a reduction in O-GlcNAc modification of STAT6, which inhibited the immune-suppressive program of M2 macrophages. Furthermore, the study demonstrated that M2 macrophages, which require high metabolic demands, reprogram their metabolic and bio-energetic pathways from fatty acid oxidation to glycolysis (40). The above studies hint that immune-metabolic regulation of O-GlcNAcylation in macrophages may have the prospect of clinical application.

## O-GlcNAcylation in dendritic cells

Dendritic cells (DC) recognize pathogens in innate immunity and activate immune cells in adaptive immunity (41). DC maintain peripheral T cells in a static state in the absence of inflammatory stimuli and identify signals associated with pathogens or inflammation continually (42, 43). If pattern recognition receptors (PRRs) on DC recognize pathogen-associated molecular patterns (PAMPs) or damage-associated molecular patterns (DAMPs), DC become activated or mature (44, 45). DC can express a broad range of PRRs, including surface and intra-nuclear toll-like receptors (TLRs), C-type lectins, and cytosolic sensors. After PRR activation, DC will change gene transcription, cell morphology, and cell metabolism and eventually mature and effectively activate T lymphocytes (44). Gibec D et al. suggested the impact of O-GlcNAcylation in human monocyte-derived dendritic cells (moDCs). They found that the reduction of OGT affected the AKT and MEK/ERK signaling pathways in moDCs and resulted in alterations to the maturation process of immature moDCs. Simultaneously, they observed that HLA-DR, CD14, and CD86 expression increased. In contrast, the expression of CD80 and DC-SIGN was reduced (46). A recent study showed that inhibiting OGA promotes the differentiation of CD34+ hematopoietic stem and progenitor cells (HSPCs) and acute myeloid leukemia (AML) cells into DC *via* STAT3/5 signaling (47). Further, cullin family E3 ubiquitin ligase (CUL3) reduces expression of OGT, leading to inhibition of STAT3 O-GlcNAcylation. However, the current studies about O-GlcNAcylation in dendritic cells remain insufficient. At present, studies on O-GlcNAcylation in dendritic cells are still lacking, and more studies are needed to clarify the function of O-GlcNAcylation in dendritic cells in the future.

## O-GlcNAcylation in neutrophils

Neutrophils are polymorphonuclear granulocytes (PMNs) derived from bone marrow stem cells (48, 49). Neutrophils phagocytose and kill microorganisms by participating in the inflammatory response and cell apoptosis (50, 51). Some hold the opinion that neutrophils are at the crossroads of innate and adaptive immunity (52). Previous studies indicated that the level of proteins in neutrophils that undergo O-GlcNAcylation increased rapidly within 2 minutes after exposure to N-formylmethionine-leucyl-phenylalanine (53). Marchase RB and Kneass ZT reported that protein O-GlcNAcylation in PMNs could be significantly increased by both glucosamine (GlcN) and agonist, with the two treatments displaying similar and additive effects (54). Some studies showed that substrates or inhibitors increase the overall O-GlcNAcylation level and promote the activation of p38 and ERK in neutrophils. Elevated O-GlcNAcylation levels enhanced neutrophil migration and motility (55). Meanwhile, the administration of PUGNAc (OGA inhibitor) or GlcN leads to the upregulation of Rac activity. GlcN and PUGNAc increase both the basal and fMLP-induced activity of a central mediator of cellular motility, the small GTPase Rac (56). Rac has been shown to be crucial in regulating the mobilization of neutrophils and activating downstream signaling pathways, such as p38 and p44/42 MAPK signaling (55–57). To sum up, O-GlcNAcylation facilitates neutrophil mobilization by increasing Rac activation and is a process of dynamic change in neutrophils. Moreover, this dynamic change may reflect some pathophysiologic status in immune related disease.

## O-GlcNAcylation in natural killer cells

As part of the innate immune system’s effector lymphocytes, Natural killer (NK) cells serve to control the growth of tumors and microbial infections by restricting their spread and minimizing accompanying tissue damage (58). NK cells, accounting for around 15% of circulating lymphocytes, are considered the third primary type of lymphocyte, alongside T cells and B cells (59). NK cells were a crucial component of the innate immune system, mainly distributed in the peripheral blood, liver and spleen (60). *In vivo*, NK cells were mostly identified by the absence of CD4 and the presence of CD56 (61). GST-sHLA-G1α chain could inhibit the alteration of O-GlcNAc levels in NK cells. One study indicated that GST-sHLA-G1a chain could bind to its receptor ILT-2 on NK92 cells and recruited SHP-1, which consequently dephosphorylated some important protein tyrosine kinases (PTK) and blocked the activation of MEK and ERK (62). Reshmi et al suggested that exposing NK cells to the cytokines IL-2 and IL-15 resulted in heightened O-GlcNAcylation of various cellular proteins (63). When the O-GlcNAcylation of NK cells was inhibited by the chemical molecule OSMI-1, the expression of NKG2D, NKG2A, NKp44, TNF-α, IFN-γ, granulysin, soluble Fas ligand, perforin, and granzyme B were decreased. This resulted in a reduction of NK cell cytotoxicity against cancer cells. However, when OGA was inhibited, and O-GlcNAcylation was increased, there was no effect on NK cell cytotoxicity. Experiments conducted *in vivo* revealed that pretreatment with OSMI-1, an inhibitor of O-GlcNAcylation, resulted in a reduction in the cytotoxic activity of NK cells against tumor cells (63). Upon NK cell stimulation, NFAT has been proved to relocate from the cytosol to the nucleus (64). The impact of O-GlcNAcylation of NFAT on NK cell function remains uncertain. Therefore, further investigation is needed to explore the plausible role of O-GlcNAcylated variants of NF-κB and NFAT transcription factors in regulating the cytotoxic capabilities of NK cells. By inhibiting the activity of enhancer of zest homolog 2 (EZH2)’s histone methyl transferase; progenitor cells can be differentiated into mature NK cells. In addition, O-GlcNAcylation of EZH2 at serine 75 regulates NK cell development and EZH2 protein stability and function (65, 66). O-GlcNAcylation is crucial in preventing c-Myc clearance and decreasing UDP-GlcNAc levels due to glutamine depletion (67). NK cells are lymphocytes with anti-tumor functions and the cytotoxicity depends on c-Myc. Therefore, the cytotoxic activity of NK cells against tumor cells may contribute to the immunotherapy of cancer.

## Adaptive immunity

Adaptive immunity, also known as specific immunity or acquired immunity, is the immune response that is developed by an organism after exposure to specific antigens (68). Adaptive immunity has developed a sophisticated system of recognition for both self-antigens and nonself-antigens, allowing for a broader and more precise immune response (69). It is generally formed after the stimulation of antigenic substances such as microorganisms (immunoglobulins, immune lymphocytes), and can react specifically with the antigen. T and B cells are the major components of acquired immunity (70). Guerini D et al demonstrated the crucial involvement of OGT in the activation of T and B lymphocytes (71). Proper proliferation and development of most immune cells rely on sufficient O-GlcNAcylation. Decreased O-GlcNAcylation of NF-κB, NFAT, and c-Myc in T and B cells, as well as NF-κB in macrophages, results in loss of their function (72, 73).

## O-GlcNAcylation in T cells

T lymphocytes (T cells), also known as thymus-dependent lymphocytes, are the main components of lymphocytes. T cells orchestrate various facets of adaptive immunity, encompassing reactions against pathogens, allergens, and cancer cells (74). After experiencing hematopoietic cell stress caused by chemotherapy, radiotherapy, infection, or transplantation, the extent of immune reconstitution, particularly of T cells, is closely related to patient outcomes (75). The process of T cell activation is reliant on O-GlcNAcylation and this effect is partially attributable to the O-GlcNAcylation of NF-κB and NFAT, which promotes their translocation from the cytoplasm to the nucleus and drives the proliferative response (71). The differentiation of T helper cells, including Th1, Th2, and Th17 cells, depend crucially on O-GlcNAcylation of NFAT (76). During the initial stages of T-cell activation, there is a notable surge in O-GlcNAcylation levels of nuclear proteins, while O-GlcNAcylation on specific cytosolic proteins swiftly declines (77). Elevated levels of O-GlcNAcylation promoted pro-inflammatory CD4^+^ differentiation and pro-inflammatory Th1 and Th17 effector cells are recognized to be augmented in situations of altered metabolism, such as type 1 and 2 diabetes and inflammatory bowel disease (73). O-GlcNAcylation is activated in primary human T cells. OGT but not OGA function is linked to OGT localization and downstream of TCR signaling. Furthermore, LCK and ZAP-70 were identified as new O-GlcNAcylated proteins among these proximal kinases (78). Several studies hinted at the function of O-GlcNAc in CD4^+^ T cell differentiation in autoimmune diseases. In systemic lupus erythematosus, T cells in female patients display elevated levels of OGT expression and OGT demethylation (79). Cantrell DA et al. suggested that the presence of c-Myc is essential for the maintenance of protein O-GlcNAcylation in T cells. Loss of OGT impeded the renewal of T cell progenitors, malignant transformation, and clonal expansion of peripheral T cells (80). Additionally, in T cells of patients diagnosed with multiple sclerosis, reduced expression of miR-15-b upstream of OGT leads to increased Th17 cell differentiation (81). Peng Wu et al. showed that the O-GlcNAc level was elevated *in vitro* differentiated effector CD8+ T cells and *in vivo* activated effector CD8+ T cells, as well as *in vitro* differentiated memory-like CD8+ T cells (82). Many O-GlcNAcylation proteins are newly identified in T cell subsets, so further investigation is necessary to fully understand the exact mechanisms through which O-GlcNAc impacts T-cell biology.

## O-GlcNAcylation in B cells

B cells are one type of lymphocyte mainly derived from bone marrow (83). B cells are the main cells of humoral immunity, which mainly produce immune effects by secreting antibodies (84, 85). Hyper-O-GlcNAcylation activates NF-κB signaling through crosstalk with phosphorylation and acetylation. O-GlcNAcylation of p65 at Thr-305 and Ser-319 can increase CREB-binding protein (CBP)/p300-dependent by activating acetylation of p65 at Lys-310, and contribute to NF-κB transcriptional activation (86). Upon B-cell receptor (BCR) cross-linking, O-GlcNAcylation is increased. O-GlcNAcylation of lymphocyte-specific protein-1 (Lsp1) in activated B cells is a crucial determinant that initiates apoptosis. The O-GlcNAcylation of Lsp1 at S209 is necessary for the recruitment of PKC-β1, which subsequently triggers S243 phosphorylation, resulting in ERK activation and the reduction of BCL-xL and BCL-2 (87). Lin KI et al. suggested that lack of Ogt could enhance mature B cell apoptosis, thus disrupting the homeostasis of B cells. Meanwhile, Lyn O-GlcNAcylation at serine 19 is essential for BCR-mediated B cell activation (88). Zhang B et al. indicated that the O-GlcNAcylation in pre-B acute lymphocytic leukemia exacerbates the progression of the disease through PI3K/Akt/c-Myc pathway (89). Park SK et al. showed that enhanced O-GlcNAcylation of c-myc facilitates the proliferation of pre-B cells by upregulating the expression of A-type and E-type cyclins. Through overexpression of c-Myc in insect cells, they provided evidence that O-GlcNAc occurs at c-Myc threonine 58, a frequently mutated hot spot in human lymphomas (90, 91). These results indicated that O-GlcNAcylation-dependent expression of c-Myc might be a potential therapeutic target for pre-B cell-derived leukemia. Overall, O-GlcNAcylation is crucial for maintaining various types of B cells and plasma cells.

## O-GlcNAcylation in disease

### O-GlcNAcylation in cardiovascular diseases

Cardiovascular disease is a group of circulatory system diseases mainly characterized by heart and cardiovascular abnormalities, among which the common ones are hypertension, myocardial infarction, myocardial hypertrophy, diabetic heart disease, etc. Increased O-GlcNAcylation of cardiomyocytes has been reported in various cardiovascular diseases. Umapathi P et al. showed that lowering O-GlcNAcylation prevented myocardial hypertrophy in mice after TAC surgery, and increasing O-GlcNAcylation induced progressive cardiomyopathy in mice (92). Kronlage M et al. suggested that histone deacetylase 4 (HDAC4) plays a protective role in diabetic hearts by knocking it down in cardiomyocytes of diabetic mice. They further found that the N-terminal protein fragment of HDAC4 is a key factor in the cardioprotective effect and that the O-GlcNAcylization at serine-632 is an important step in the production of the N-terminal protein of HDAC4 (93). Ischemia-reperfusion (I/R) injury is a complex problem in treating myocardial infarction. Ou W et al. reported that hypoxic acclimation enhanced antioxidant effects by activating G6P dehydrogenase through O-glcnacylation in the heart; thereby protecting cardiomyocyte mitochondria and reducing I/R damage (94).

### O-GlcNAcylation in cancer

In recent years, the regulatory role of O-GlcNAcylation in cancer has received extensive attention. The redistribution of glucose utilization is one of the essential links in cancer. Shi Q et al. showed that M2 tumor-associated macrophages have the highest glucose uptake capacity in tumors and promote O-GlcNAcylation in the hexosamine biosynthesis pathway, eventually promoting tumor metastasis and drug resistance (95). Wang X et al. reported that glucose-induced O-GlcNAcylation promotes ferroptosis in mesenchymal pancreatic cancer cells. Further, they demonstrated that ZEB1, mesenchymal property transcription factor, O-GlcNAcylation at Ser555 promotes glucose-driven iron death in mesenchymal pancreatic cancer cells (96). Lee DE et al. indicated that inhibition of O-GlcNAcylation can promote autophagy of HCT116 cells of colon cancer, while metformin can induce endoplasmic reticulum stress of HCT116 cells to enhance autophagy. Their follow-up study found that treatment of HCT116 cells with a combination of metformin and OSMI-1 led to sustained induction of autophagy, which in turn enhanced apoptosis through a synergistic effect (97).

## Conclusions

Protein O-GlcNAcylation regulates several immune signaling pathways, such as NF-κB, mTOR/S6K1, ERK, FOXO1, CPT2, PGC-1α, JNK, and p38, MAPK. Protein O-GlcNAcylation is closely related to the activation of immune signaling and inflammatory responses. An expanding body of research has revealed O-GlcNAcylation as a vital homeostatic regulator at the intersection of inflammation and metabolism which extensively engaged in cardiovascular diseases and cancer. More and more evidence proved a direct correlation between energy metabolism and O-GlcNAcylation and O-GlcNAcylation plays a dual role of protection and damage in diseases. These suggest that targeting O-GlcNAcylation may have therapeutic potential for treating cancer, cardiovascular diseases, and other immune-related disorders. The implications of O-GlcNAc signaling in immune regulation, particularly in the context of metabolic perturbations, may be far-reaching.

## Author contributions

HC and WX wrote the initial draft of the manuscript. HZ prepared the figure and revised the manuscript. QW conducted the literature search. ZL and SL revised and contributed to the final draft. All authors have contribution to the article and approved it for publication.
